# From invasive to non-invasive: point-of-care ultrasound parameters and a clinical decision algorithm for central venous pressure assessment in critically Ill neonates

**DOI:** 10.3389/fped.2026.1871758

**Published:** 2026-09-22

**Authors:** Runshu Zhang, Rong Ju, Yiyong Fu, Xinyu Lin, Xin Liu, Qun Huang, Yajun Huang

**Affiliations:** 1 Department of Pediatrics, The Affiliated Hospital of Southwest Medical University, Luzhou, Sichuan, China; 2 Department of Pediatrics, Chengdu Wenjiang District Maternal and Child Health Hospital, Chengdu, Sichuan, China; 3 Department of Neonatology, Chengdu Women’s and Children’s Central Hospital, School of Medicine, University of Electronic Science and Technology of China, Chengdu, Sichuan, China; 4 Department of Pediatrics, 363 Hospital, Chengdu, Sichuan, China

**Keywords:** point-of-care ultrasound, central venous pressure, neonate, hemodynamic monitoring, inferior vena cava, collapsibility index, fluid resuscitation, critical care

## Abstract

Point-of-care ultrasound (POCUS) is increasingly used in neonatal intensive care units (NICUs) for hemodynamic assessment, but standardized protocols for central venous pressure (CVP) estimation remain lacking. This narrative review synthesizes current evidence on POCUS parameters—primarily the inferior vena cava collapsibility index (IVC-CI), maximum IVC diameter, the inferior vena cava-to-aortic ratio (IVC/Ao), and internal jugular vein (IJV) indices—for non-invasive CVP assessment in critically ill neonates. Key advances include demonstrating that ultrasound-measured IVC diameter correlates with invasively measured CVP in low birth weight infants and the emergence of multi-parameter models. However, major barriers persist: inconsistent neonatal-specific thresholds, operator dependence, limited training infrastructure, and insufficient validation in extremely low birth weight populations. We propose a practical clinical decision algorithm integrating IVC and IJV parameters with adjunctive lung and cardiac POCUS assessment, structured reassessment after every fluid bolus, and objective triggers for transitioning to invasive monitoring. Recent guidelines from the European Society of Paediatric and Neonatal Intensive Care (ESPNIC) and the American Society of Echocardiography (ASE) provide foundational frameworks, but dedicated neonatal training and credentialing standards remain essential. Future directions include artificial intelligence-assisted measurement, multi-parameter scoring systems (e.g., Venous Excess Ultrasound score), and multicenter prospective validation. POCUS serves as a complementary tool to—not a replacement for—invasive monitoring in complex hemodynamic instability. Standardized thresholds, structured training, and quality assurance programs are critical for safe clinical translation, especially in resource-limited settings.

## Highlights

- POCUS is a safe, non-invasive first-line tool for CVP assessment in stable critically ill neonates.
- IVC-CI is the most validated parameter; IJV assessment is reserved as an adjunct when IVC imaging is inadequate.
- Tentative neonatal IVC-CI stratification: <15% suggests venous congestion; 15%–30% presumptive euvolemia; 30%–50% an indeterminate zone requiring adjunctive IJV, lung, or cardiac POCUS confirmation; >50% probable hypovolemia warranting a 5–10 mL/kg fluid challenge followed by structured reassessment.
- POCUS reduces unnecessary CVC insertion and lowers CRBSI risk, especially beneficial in resource-limited settings.
- POCUS complements—does not replace—invasive monitoring in unstable or complex hemodynamic conditions.
- Structured training and quality assurance programs are essential for accurate and safe POCUS implementation.

## Introduction

Central venous pressure (CVP) is a cornerstone hemodynamic parameter that reflects right atrial pressure, intravascular volume status, venous compliance, and cardiac function, guiding fluid resuscitation and hemodynamic management in critically ill neonates (1). Typically, lower CVP values suggest hypovolemia or dehydration, whereas higher CVP values indicate hypervolemia or congestive heart failure. This vulnerable population—including premature infants, very low birth weight infants (VLBWIs), and those with septic shock, respiratory distress syndrome (RDS), and congenital heart disease (CHD)—exhibits immature cardiovascular physiology and heightened susceptibility to hemodynamic instability (2, 3).

Invasive CVP monitoring via central venous catheterization (CVC) remains the gold standard but carries substantial risks: catheter-related bloodstream infections (CRBSIs), vascular injury, thrombosis, malposition, and cardiac tamponade (1, 2). A scoping review by Comber et al. identified high rates of implementation failures in neonatal central venous access device practices (2). Moreover, invasive monitoring is resource-intensive, requires specialized expertise, and cannot be performed dynamically at the bedside, limiting its utility in emergency neonatal care (1, 4).

Point-of-care ultrasound (POCUS)—non-invasive, real-time, repeatable, and bedside-accessible—has emerged as a promising alternative for CVP assessment (3, 5). By measuring central vein diameter, collapsibility, dynamic changes, and the ratio of inferior vena cava to aortic diameter, POCUS enables non-invasive evaluation of right-sided filling pressures (5, 6). In their systematic review of critically ill children, Hakim et al. documented moderate-to-strong correlations between these venous indices and invasive CVP; notably, only one included study enrolled neonates—the echocardiographic work by Mugloo et al.—which reported a strong correlation between CVP and IVC-CI independent of gestational age or birth weight (5, 7). The American Society of Echocardiography updated its guidance in 2024, endorsing cardiac point-of-care ultrasound to inform volume resuscitation and ventilator strategy when impaired venous return contributes to instability, while cautioning that fetal shunts complicate volume-status assessment and that the relationship between IVC size, collapsibility, and volume change remains incompletely established in neonates, particularly during invasive ventilation (8). The Journal of Perinatology has played a pioneering role in neonatal POCUS, publishing one of the first case reports on portable ultrasound for diagnosing intraventricular hemorrhage in 2002 (9). Recent publications have further advanced the field, including a scoping review of Neonatal SONography Assisted Resuscitation (NeoSONAR) and guidelines for neonatal POCUS training, credentialing, and quality assurance (10–14).

Nevertheless, current research remains constrained by inconsistent neonatal-specific thresholds, single-center small-sample designs, operator dependence, and insufficient validation in special populations (11, 15). This narrative review synthesizes current evidence on POCUS parameters for non-invasive CVP assessment in critically ill neonates, proposes a practical bedside decision algorithm anchored to explicit exclusion criteria, and outlines how we intend to validate and implement it.

## Literature search strategy

Because this article is designed as a narrative (non-systematic) review, a targeted rather than exhaustive literature search was conducted in PubMed, PMC, and Web of Science for articles published between January 2021 and March 2026 using keywords combining “point-of-care ultrasound” or “POCUS” with “central venous pressure” or “CVP” and “neonate,” “newborn,” “infant,” “premature,” or “VLBWI.” Additional terms included “inferior vena cava,” “internal jugular vein,” “collapsibility index,” and “hemodynamic monitoring.” Priority was given to prospective cohort studies, randomized controlled trials, and clinical guidelines; systematic reviews were consulted chiefly to identify relevant primary neonatal studies rather than to define the scope of this work. No protocol registration, systematic screening, or meta-analysis was undertaken, consistent with the narrative design of this review. Reference lists of included articles were also screened for additional relevant studies.

## Core POCUS parameters for non-invasive CVP assessment

POCUS assesses CVP by measuring anatomical and dynamic changes of central veins closely related to right atrial pressure and intravascular volume (5, 6). The most validated parameters include IVC indices, IJV indices, and supplementary functional parameters.

## Inferior vena cava (IVC) parameters

The IVC directly connects to the right atrium, making its diameter and collapsibility closely coupled with right atrial pressure (5, 7). By convention, IVC-CI indexes variation of IVC diameter across the respiratory cycle, calculated as [(IVCDmax−IVCDmin)/IVCDmax] × 100% (5, 16). Deliberate sampling of inspiratory maxima and expiratory minima is straightforward in cooperative adults and older children; in newborns, rapid irregular breathing and the inability to coordinate respiratory effort make phase-matched timing difficult or impossible, so most neonatal investigations have computed the index from observed extremes without regard to respiratory phase (5, 7). A systematic review by Hakim et al. in critically ill children demonstrated moderate to strong correlations between IVC-CI and invasive CVP (r = 0.58–0.73) (5), and the comprehensive systematic review and meta-analysis by Shaban et al. confirmed that ultrasound-derived IVC indices provide useful CVP estimates across various patient populations, including children (6). Both reviews emphasized substantial heterogeneity among the included studies—small samples, mixed ventilation status, varied acquisition protocols—and concluded that larger, higher-quality validation studies are required before published cut-offs can be generalized (5, 6).

Absolute diameters tell a more complicated story. In neonates, Mugloo et al. found that neither IVCDmin nor IVCDmax tracked CVP; both diameters instead scaled with gestational age and birth weight (7). Sato et al. described the same dependence of the individual diameters on gestational age and weight, yet observed that the relationship between them—minimum expressed against maximum—was almost independent of either and correlated strongly with CVP (16). More recently, Li et al. demonstrated a significant correlation between ultrasound-measured IVC diameter and CVP obtained through peripherally inserted central catheters in low birth weight infants (17). Absolute IVC measurements also shift appreciably over the early postnatal period: in preterm neonates, median maximum IVC diameter rose from day one through day three before falling back toward the close of the first week, so serial interpretation should be anchored to day of life (18).

A further pragmatic option is the inferior vena cava-to-aortic ratio (IVC/Ao). Within the same subcostal sweep, the IVC is measured at end-expiration in its intrahepatic segment while the aorta is measured during systolic ventricular ejection (19). In critically ill dehydrated children, Babaie et al. reported a moderate positive correlation between IVC/Ao and CVP (r = 0.42), with lower ratios discriminating reduced intravascular volume reasonably well (19); synthesized experience summarized in the Hakim review proposes ratios below approximately 0.8 to signal inadequate preload and the need for fluid, vs. values above roughly 1.2 suggesting overhydration (5). Because both calibers are captured within a single quiet window, the ratio is less dependent on brisk respiratory collapse—an attractive property in newborns whose breathing effort varies moment to moment—although normative neonatal values remain scarce and combined caval-aortic measurement has so far been evaluated mainly in observational pediatric series (10).

IVC parameters remain vulnerable to abdominal distension and positive end-expiratory pressure (PEEP), which may cause false-low CVP estimations (5). Sato et al. also noted that IVC diameter accuracy is influenced by respiratory support settings (16). The mechanical transmission of PEEP to central veins, however, is partly buffered: when lung compliance is normal, no more than about a quarter of the applied airway pressure reaches the pleural space and the central venous compartment, and the compensatory increase in mean systemic filling pressure preserves venous return and cardiac output (20). Provided the infant is clinically stable, ventilator settings are documented beside the scan, and collapsibility margins are interpreted conservatively, IVC-CI therefore remains interpretable even under modest PEEP; caution concentrates on high-support, low-compliance lungs (20).

## Internal jugular vein (IJV) parameters

The IJV provides an alternative when IVC imaging is limited, being less affected by abdominal conditions (21). IJV diameter and IJV collapsibility have been studied as potential volume-status markers. Chayapinun et al. highlighted the utility of IJV evaluation when IVC windows are suboptimal (21), and Jaffe et al. demonstrated that force-coupled ultrasound of the IJV enables non-invasive CVP estimation with reasonable accuracy (22). Two physiological caveats temper enthusiasm, however. First, cerebral autoregulation actively modulates jugular blood volume: shifts in arterial carbon dioxide tension, systemic blood pressure, posture, and cerebral metabolic demand alter IJV caliber independently of right-sided filling pressures, decoupling jugular indices from CVP in ways that cannot be reliably anticipated (21). Second, to our knowledge no study has evaluated the feasibility, reproducibility, or reference ranges of IJV-based CVP indices specifically in neonates. Pediatric evidence remains mixed. A prospective study by Jayasimha et al. in critically ill postoperative pediatric cardiac patients found no significant correlation between CVP and either the IJV/common carotid artery ratio or the IJV distensibility index, suggesting limited utility for IJV POCUS in that population (23). Thus, IJV parameters should be considered adjunctive rather than primary in neonatal CVP assessment until dedicated neonatal data become available.

## Other supplementary parameters

Hepatic vein Doppler flow patterns provide supplementary value: Franzon et al. showed that hepatic vein Doppler can estimate CVP in mechanically ventilated children (24), although smaller hepatic vein diameters and higher, irregular respiratory rates may affect Doppler signal acquisition in neonates. Right ventricular functional parameters (RV fractional area change, TAPSE) contribute additional context on the pumping side of the circulation (25). Right ventricular performance, however, cannot be meaningfully reduced to a single linear excursion. Evaluation is materially richer than TAPSE alone—especially in complex physiology such as pulmonary hypertension or congenital diaphragmatic hernia, where septal geometry, persistent fetal shunts, and abnormal loading conditions coexist (8). Within cardiac POCUS, hyperdynamic systolic function of the left ventricle and mid-cavitary flow acceleration offer additive clues to an adrenergic, underfilled circulation, whereas overt RV dilation or dysfunction should redirect the clinician toward targeted neonatal echocardiography with comprehensive functional assessment rather than isolated parametric readings (8, 25, 26). Elsewhere, whenever findings diverge from the clinical trajectory, full functional echocardiography remains indicated (8).

## Comparison of IVC and IJV parameters

The heterogeneity in reported correlation coefficients reflects differences in patient populations, ventilation settings, and measurement techniques (3, 6). Most of that evidence derives from older pediatric cohorts, and the scarcity of neonatal-specific validation argues against transplanting established cut-offs downward without re-testing them in this age group (5, 7). This variability underscores the need for multi-parameter approaches (3, 6).

## Physiological mechanisms in neonates

The correlation between POCUS parameters and CVP in neonates is strongly modulated by unique cardiovascular physiology (1, 7). Neonates have lower central venous compliance due to immature vascular smooth muscle, making venous indices more sensitive to small volume changes (7). Immature right ventricular function further disrupts the relationship between venous collapsibility and filling pressure (25).

Respiratory pattern, PEEP, sedation, and abdominal distension independently affect POCUS measurement reliability, explaining the heterogeneity of threshold values across studies (15, 16, 21). Postnatal maturation of caval geometry itself adds a time-dependent component to every longitudinal reading (18).

## Clinical applications

### Guidance of fluid resuscitation

Framed practically, the value of these measures lies in using CVP-mirroring venous behavior to assess intravascular volume status—real-time, repeatable information on which fluid management and corrective therapeutic strategies can be built (3, 5).

POCUS-guided fluid stewardship addresses both over-resuscitation and under-resuscitation. In neonatal septic shock, a randomized controlled trial demonstrated better clinical outcomes when bolus decisions were guided by lung ultrasound rather than routine management, positioning ultrasound not merely as a surveillance instrument but as an active titration aid (27). Why structured reassessment matters is illustrated by cardiac-index evidence: among children receiving boluses for septic shock, Long et al. found that responses in cardiac index varied widely and could not be taken for granted—a reminder that fluid responsiveness should be verified after every aliquot rather than assumed (28).

### Hemodynamic monitoring in special populations

POCUS offers clear advantages in VLBWIs and CHD neonates, who face high risks from invasive catheterization (2, 17). Mi et al. described abnormal IVC and abdominal aorta characteristics in neonates with early-onset septic shock, supporting POCUS for early hemodynamic assessment (29).

### Prevention of invasive monitoring complications

By reducing unnecessary CVC insertion, POCUS decreases CRBSI, vascular injury, and thrombosis rates (2, 3), with greatest benefit in preterm and clinically unstable neonates (2, 4).

### Clinical decision algorithm

Figure 1 condenses the recommended pathway into three phases. Patient selection first removes the physiologies for which simplified venous reasoning fails: ECMO, complex single-ventricle anatomy, CHD, CDH, acute pulmonary hypertension, and refractory shock, operationalized here as hypoperfusion persisting despite adequate volume resuscitation together with an escalating vasopressor requirement and lactate > 4 mmol/L. For the remaining infants, IVC acquisition follows a fixed recipe—supine position, subcostal long-axis view, an M-mode cursor crossing the intrahepatic IVC roughly 3–4 cm caudal to the caval–right atrial junction, and millimeter-recorded diameters sampled across several unforced breaths (7). Collapsibility bands then steer therapy: below 15% congestion prompts restriction or diuresis; 15%–30% presumptive euvolemia continues scheduled observation; the 30%–50% band is treated as indeterminate and defers any bolus until adjunctive IJV, lung, or cardiac POCUS findings corroborate the impression; and collapse exceeding 50%, consistent with the threshold favored in current reviews, triggers a 5–10 mL/kg fluid challenge (5). Every bolus obliges structured reassessment—repeat IVC-CI supplemented by lung and cardiac POCUS—to confirm ongoing responsiveness before proceeding, while explicit deterioration criteria govern escalation to ultrasound-guided central cannulation; conversely, infants already carrying a catheter graduate to removal after a stable day and two consecutive concordant scans (27). Acquisition technique, strengths, and limitations of each parameter are consolidated in Table 1.

**Figure 1:**
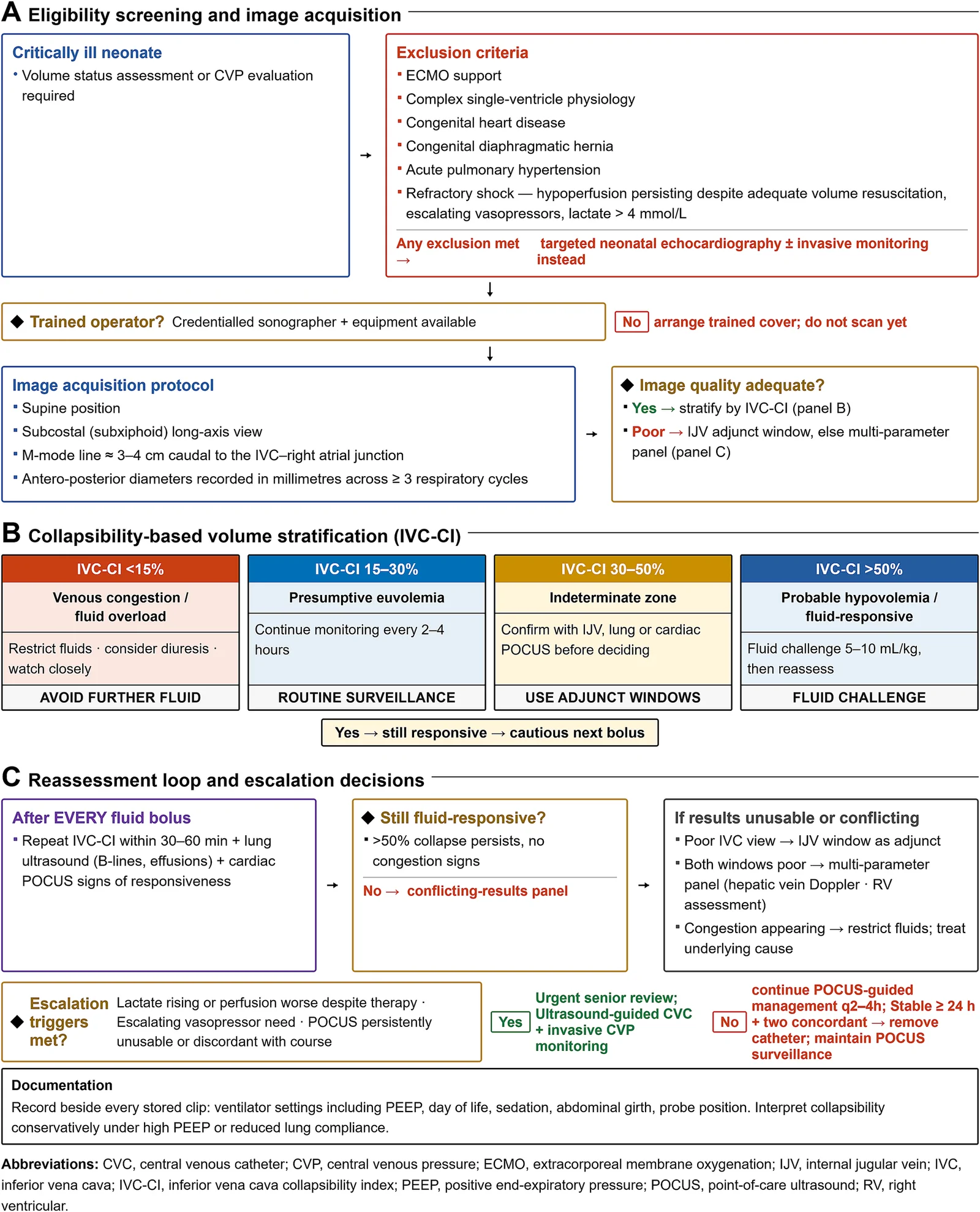
| Clinical decision algorithm for non-invasive central venous pressure assessment in critically ill neonates. Eligibility screening incorporates the expanded exclusion physiologies, image acquisition follows the standardized subcostal recipe, collapsibility stratification includes a deliberately indeterminate 30%–50% zone, and the lower lane carries the post-bolus reassessment loop and escalation triggers.

**Table 1:** Practical summary of POCUS parameters for non-invasive CVP assessment in critically ill neonates.

Parameter	How images are acquired	Strengths	Limitations
Maximal/minimal IVC diameter	Supine; subcostal long-axis view; M-mode cursor ≈ 3–4 cm caudal to the IVC–right atrial junction; antero-posterior diameters recorded in millimetres across ≥ 3 respiratory cycles	Simplest static index; validated against PICC-sampled CVP in low-birth-weight infants; tracks gestational-age and weight trajectories	Absolute values confounded by gestational age, birth weight, and rapid early-postnatal change; cannot be compared across infants without age anchoring
IVC-CI	Same window; record maximal and minimal antero-posterior diameters; index = [(IVCDmax−IVCDmin)/IVCDmax] × 100%; where phase-matching is impossible, use observed extremes over ≥ 3 unforced breaths	Most studied neonatal index; correlation with CVP reported independent of gestational age and birth weight; quick bedside signal	Requires genuine respiratory variation — blunted by crying, high-frequency ventilation, PEEP, sedation; published thresholds vary and await prospective calibration
IVC/Ao ratio	One quiet subcostal loop; IVC measured end-expiration in the intrahepatic segment; aorta measured during systolic ejection; ratio derived from the single matched pair	Captured within a single window; less dependent on vigorous respiratory collapse; practical screening property	Fewer validation data than IVC-CI; evidence mainly from older paediatric cohorts; neonatal reference ranges sparse
IJV diameter/collapsibility	High-frequency linear probe; transverse neck at mid-thyroid cartilage level; light probe contact without compression; maximal and minimal antero-posterior diameters analogous to IVC	Accessible when the abdomen is unavailable (distension, dressings, surgical sites); non-ionising and repeatable	Calibre modulated by cerebral autoregulation, head position, intrathoracic pressure; transducer-pressure artefact; no neonatal feasibility or reference-range studies
Hepatic vein Doppler	Subcostal views; pulsed-wave sample at the hepatic vein–IVC confluence; analyse systolic/diastolic waves and any atrial-reversal component across ≥ 5 cardiac cycles	Infers pressure from flow morphology rather than calibre alone; applicable during mechanical ventilation	Small hepatic veins and fast irregular breathing degrade signals; expertise-dependent; minimal neonatal experience
RV functional measures	Apical four-chamber window; TAPSE by M-mode at the lateral tricuspid annulus; fractional area change from traced diastolic and systolic areas; LV shortening fraction ± mid-cavitary velocity–time integral	Places venous readings in context of pump function and afterload; detects pulmonary-hypertension signatures	Not direct CVP surrogates; TAPSE alone inadequate in complex physiology (pulmonary hypertension, CDH); abnormal findings mandate targeted neonatal echocardiography

## Discussion

The shift from invasive to non-invasive CVP assessment using POCUS represents a major advance in neonatal critical care, particularly for vulnerable populations in NICUs worldwide. For preterm and critically ill neonates, avoiding unnecessary CVC placement directly reduces life-threatening complications such as CRBSI, vascular injury, and thrombosis, which are more prevalent in low-birth-weight and clinically unstable infants (1, 2, 4). This review confirms that IVC and IJV ultrasound parameters are feasible, reproducible, and moderately to strongly correlated with invasive CVP measurements, supporting their role in routine bedside hemodynamic assessment.

A notable strength of current evidence is the significant correlation demonstrated between ultrasonographically measured IVC diameter and CVP sampled through peripherally inserted central catheters in low birth weight infants, a group in whom every avoided procedure carries disproportionate benefit (17). The consistent correlation between IVC parameters and CVP in neonates provides a solid physiological basis for clinical application (5–7, 16). Meanwhile, the IJV serves as a reliable alternative when abdominal conditions impair IVC visualization, expanding the applicability of POCUS in sick neonates (21, 23).

Foremost among unresolved issues is the absence of settled neonatal thresholds for IVC-CI. Published cut-off values range widely—from 25% to 50%—reflecting variations in gestational age, ventilation status, and measurement methodology (5, 7). Traditional practice anchors probable hypovolemia near a 50% collapse, a convention best supported in older children and adults (5); the present revision adopts that anchor rather than the more liberal 30% trigger proposed in our earlier draft. Acknowledging that newborns generate shallower excursions yet declining to discard the available literature, the algorithm now classifies collapse beyond 50% as probable hypovolemia, reserves 30%–50% as an indeterminate zone requiring adjunctive IJV, lung, or cardiac confirmation before fluids, holds 15%–30% as presumptive euvolemia, and treats collapse under 15% as congestion. These boundaries remain proposals that require large-scale prospective calibration rather than validated constants (5, 6).

Operator dependence remains a major barrier. Studies show that measurements from inexperienced operators may differ by more than 15% from experts, leading to misclassification of volume status (10, 13). Structured training, credentialing, and quality assurance programs are therefore essential for safe implementation, yet such programs remain scarce globally (10, 11, 13, 14).

Another important gap is limited validation in extremely low birth weight infants (<1,000 g) and neonates with congenital anomalies such as abdominal wall defects or congenital diaphragmatic hernia. These infants have altered thoracic and abdominal mechanics that may significantly change IVC and IJV behavior (16, 17); they also fall outside the algorithm's screening scope by design, awaiting dedicated research (8).

The influence of positive end-expiratory pressure deserves calibrated respect rather than blanket avoidance. Each increment of applied airway pressure tends to narrow observed collapsibility and can mask hypovolemia or falsely suggest euvolemia (5). Normal-lung mechanics blunt that transmission considerably: when compliance is preserved, no more than roughly a quarter of PEEP reaches the central venous compartment, and mean systemic filling pressure rises commensurately, stabilizing cardiac output (20). Ventilator settings therefore belong beside every stored clip; collapsibility read under high support or low compliance warrants cautious interpretation, whereas stable, well-documented settings allow venous indices to retain meaning even in ventilated neonates (20).

Notably, POCUS is not a replacement for invasive CVP monitoring. In unstable neonates with complex hemodynamics—such as those post-cardiac bypass or with severe pulmonary hypertension—continuous invasive monitoring provides real-time trend data that intermittent POCUS cannot match (1, 8). Instead, POCUS functions as a frontline triage tool, a serial monitoring method, and a strategy to reduce unnecessary invasive procedures.

For resource-limited settings, POCUS is particularly valuable. It is low-cost, portable, requires no disposable supplies, and can be rapidly implemented with focused training. By reducing CVC use, POCUS lowers rates of CRBSI, a major contributor to neonatal mortality in constrained NICUs (2, 4).

How, concretely, do we intend to validate and install this algorithm? The plan proceeds in three layers. Accuracy comes first: a prospective two-center pilot will enroll consecutive eligible infants and schedule protocolized IVC-CI readings alongside routine care; wherever a central catheter is already in place, categorical POCUS judgments face their invasive counterpart directly, with agreement quantified through Bland–Altman analysis of paired pressures and weighted kappa for category concordance, and prespecified stopping rules suspending enrollment if discordance exceeds tolerance. Capability follows accuracy: operator preparation follows tiered syllabi modeled on ESPNIC guidance and recently published program blueprints—supervised proctored examinations, cumulative scan logs, and periodic image-archive audits in which a fixed fraction of stored clips undergoes blinded expert re-read (11, 13, 14). Sustainability closes the loop: implementation advances through plan-do-study-adjust cycles tracking fidelity measures (scan completion rate, image quality grades, turnaround) alongside safety counters, principally misclassified volume episodes and unplanned escalations; discrepant cases feed a standing adjudication log that continuously recalibrates operator feedback and, ultimately, threshold placement. Durable local performance would justify extension into a multicenter consortium harmonizing definitions and sharing anonymized clips through a common repository—prerequisites for any future recalibration.

Future research should focus on three priorities: (1) validation of neonatal-specific thresholds in multicenter prospective studies; (2) development of AI-assisted measurement to reduce operator dependence (30); and (3) evaluation of multi-parameter scores such as VExUS for comprehensive venous congestion assessment (3). With standardized protocols, structured training, and quality assurance, POCUS can become a standard-of-care tool in NICUs globally.

## Conclusion

POCUS is a feasible, safe, and effective non-invasive method for CVP assessment in critically ill neonates. Core IVC and IJV ultrasound parameters show moderate to high correlation with invasive CVP. Recent innovations including AI and combined parameter models have improved accuracy and reduced operator dependence. Wider clinical application requires standardized neonatal-specific thresholds, structured training programs, quality assurance frameworks, and multicenter validation studies. For resource-limited settings, POCUS provides a high-value, low-cost intervention that improves patient safety and reduces complications. With appropriate implementation, POCUS will become a standard-of-care modality for hemodynamic monitoring in NICUs worldwide.
